# Exploring the influence of lid region residues on fatty acid selectivity in a lipase originating from *Rhizopus oryzae*

**DOI:** 10.1111/febs.70284

**Published:** 2025-10-09

**Authors:** Zehui Dong, Majid Haddad Momeni, Kim Olofsson, Eva Nordberg Karlsson

**Affiliations:** ^1^ Biotechnology, Department of Chemistry Lund University Sweden; ^2^ AAK AB Malmö Sweden

**Keywords:** enzyme thermostability, lipase selectivity, structural modeling, substrate docking, *Yarrowia lipolytica* production

## Abstract

Lipases are vital in modifying lipid substrates across industries such as food, cosmetics, and pharmaceuticals. Among their features, fatty acid selectivity is particularly important for industrial applications. *Rhizopus oryzae* lipase (ROL) stands out for its high selectivity and broad applicability. In this study, we engineered single‐residue variants of ROL by targeting Ala89 and Phe95 in its lid region. Additionally, a lid‐swap chimera was created by replacing ROL's 15‐residue lid with that of the homologous lipase from *Rhizomucor miehei* (RML). These variants were expressed and characterized to assess changes in substrate selectivity. Our results highlight the lid's key role in determining fatty acid preference. Notably, mutating Phe95 to smaller residues (Ile or Ala) significantly increased selectivity toward medium‐chain fatty acid (MCFA) esters. In contrast, substituting Ala89 with bulkier residues (Phe or Trp) reduced activity—except in the lid‐swap variant. Interestingly, although the lid‐swap variant contains Trp89, the surrounding smaller, non‐conserved residues may alleviate steric hindrance. This chimera retained high activity but shifted its preference from MCFAs to long‐chain fatty acids (LCFAs), a novel observation. Overall, the engineered variants exhibited distinct substrate preferences without compromising thermostability, suggesting their potential for tailored applications in food, nutrition, and cosmetic industries.

## Graphical abstract

Lipases are vital in modifying lipid substrates across industries such as food, cosmetics, and pharmaceuticals. Among their features, fatty acid selectivity is particularly important for industrial applications. *Rhizopus oryzae* lipase (ROL) stands out for its high selectivity and broad applicability. This study aimed to engineer the substrate selectivity of ROL to fit a broader application, with protein engineering aimed at its lid region.

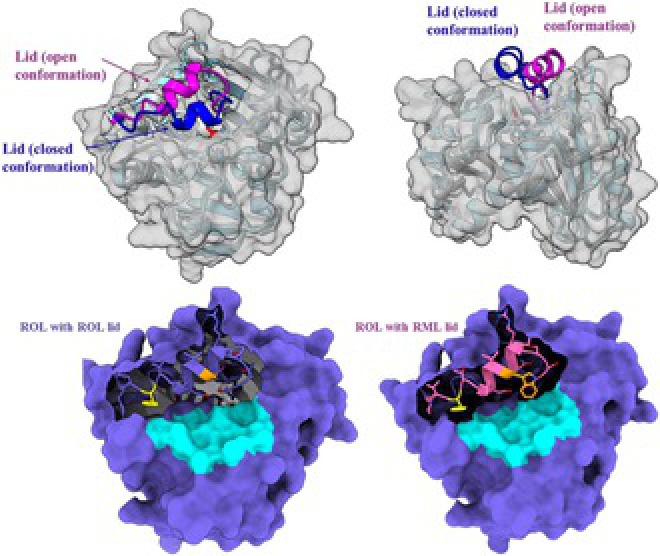

**Abbreviations**
BSA: bovine serum albumin
DSF: differential scanning fluorimetry
FA: fatty acid
LB: lysogeny broth
LCFA: long‐chain fatty acid
MCFA: medium‐chain fatty acid
MD: molecular dynamic
OD: optical cell density
*pNP*
: *p‐Nitrophenyl*
RCL: lipase from *Rhizopus chinensis*
RML: lipase from *Rhizomucor miehei*
RMSD: root mean square deviation
RMSF: root mean square fluctuations
ROL: lipase from *Rhizopus oryzae*
SCFA: short‐chain fatty acid
SDS/PAGE: sodium dodecyl sulfate‐polyacrylamide gel electrophoresis
TP: tampon phosphate
WT: wild‐type
YNB: yeast nitrogen base
YNBDL: minimal glucose medium supplemented with lysine

## Introduction

Lipases (EC3.1.1.3) are enzymes that belong to the hydrolase superfamily and are believed to originate from a common ancestor, in which all candidates possess a conserved catalytic triad (Asp (or Glu), His and Ser) [1]. Specifically, lipases are classified under the structurally diverse esterase family, which catalyze production of an acid and an alcohol in the presence of water. It is when the ester bond is connected to a mid‐ or long‐chain fatty acid (> C6) that the enzyme catalyzing its hydrolysis is called a lipase.

Lipases play a crucial role in tailoring lipid substrates (e.g., fats and oils) by catalyzing cleavage or formation of acyl compounds such as mono‐, di‐, and triglycerides, which contain mid‐ or long‐chain fatty acid (FA) esters [2, 3]. These enzymes are essential for various biological processes and are also important tools in several industrial applications, for example in oleochemical‐, food‐, detergents‐, and cosmetics industries [4, 5]. One of the essential features that makes lipases important industrial enzymes is their unique substrate specificity, evolved as a consequence of the multifarious nature of the components of the lipid substrate [6].

The substrate specificity of lipases is determined by the substrate‐binding region near the catalytic site. Most lipases have a flexible lid that covers the active site, which remains structurally closed in pure aqueous environments but opens in the presence of a hydrophobic surface. Once the lid is closed, lipases are inactive, whereas in open conformation, the catalytic center is exposed to substrates and surrounding solvents, activating the enzyme [7]. The structure of the lid domain and catalytic site are varied in lipases from different origins, resulting in enzymes with different chemo‐selectivity (preference to certain types of FA moieties), regio‐selectivity (preference for different locations on triglycerides, e.g., sn1,3 specificity), and stereoselectivity (preference between enantiomers) [8, 9].

The property of a triacylglycerol is highly depending on the FAs' composition, both concerning the FA structure and their position on the glycerol backbone. This motivates development of lipase diversity to tune activity and introduce candidates capable of modifying the FA structure, for different industrial applications. As such, the lipase from the filamentous fungus *Rhizopus oryzae* (ROL) is a promising and interesting model enzyme for lipids tailoring, due to its high regio‐selectivity toward sn1(3) positions on triacylglycerol in plant‐based fat and oil industries [10, 11]. In addition, ROL has been recombinantly produced in eukaryotic production platforms including the oleaginous yeast *Yarrowia lipolytica* [12], which is a convenient host cell factory for lipase production [13, 14]. In a previous study, we successfully constructed an efficient production cassette with erythritol inducible promoters for ROL production in *Y. lipolytica* [12], allowing genetic design and good production possibilities. Moreover, we have constructed a homology model, allowing structural comparisons of the enzyme [15].

The lid region may play an important role in lipases substrate selectivity and specificity, as shown in previous work where exchange of residues in the lids of lipases of different origin have resulted in changes in the activity profile [8, 16, 17, 18]. ROL holds a typical lid structure of one short α‐helix covering a crevice‐like substrate‐binding region located on the surface of the lipase molecule [15]. ROL has a homolog, a lipase originating from the related filamentous fungus *Rhizomucor miehei* (RML), which is another popularly used enzymes in industrial applications for lipids tailoring in industrial applications. Despite their overall structural similarity, the amino acid composition of the lid region of RML and ROL is only sharing 33% sequence identity. As site‐directed mutagenesis of some amino acid residues in the lid region of ROL has been shown to influence substrate specificity toward FAs of different length, it was interesting to replace residues in ROL to monitor the effect on specificity, especially in positions where pronounced differences with the corresponding residue in RML were present. Interestingly, Ala89 located in the center of the lid structure, important for substrate entry, is replaced by Trp at the identical location in RML, leading to a hypothesis of an unneglectable effect of this amino acid residue on substrate affinity [15]. Another residue, hypothesized to be of interest for substrate selectivity is Phe95, at the end of the lid region in close vicinity to the end of the substrate‐binding crevice [15, 19, 20]. Finally, to further investigate the function of all amino acid residues in the lid structure on substrate binding and selectivity, the complete lid‐region was swapped, which resulted in interesting effects on the substrate selectivity.

## Results

### Bioinformatic evaluation

**Fig. 1:**
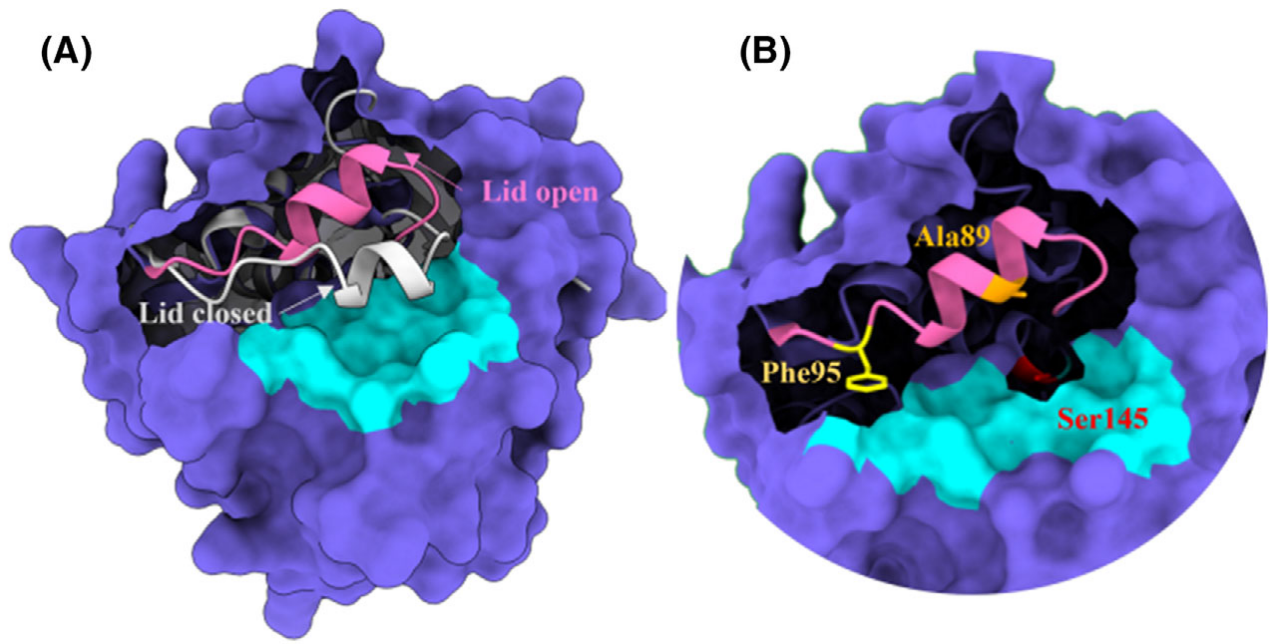
Overview of the lid structure, covering the substrate‐binding region of lipase from *Rhizopus oryzae* (ROL), in surface representation. The substrate‐binding region (shown in cyan) is covered by the lid upon closed configuration (gray helix, left structure). When the lid is open (pink helix), the substrate‐binding region and the catalytic Ser (Ser145, shown in red, right structure) are exposed to the substrate and the enzyme is activated accordingly. The structure is visualized with chimerax 1.7.1 (https://www.rbvi.ucsf.edu/chimerax).

The structure of ROL shows a typical lid, which covers both the active site and the substrate‐binding crevice during closed conformation. The designs of mutated variants, including the 15‐residue lid‐swap chimera, were implemented from the modeled structure of open‐lid ROL [8], in the active conformation, where substrate‐interacting residues are exposed to the surrounding solvent. ROL is activated upon interacting with a hydrophobic solvent or at a hydrophobic interface. The motile lid region opens like a flap and exposes the substrate‐binding region to the substrate molecules (Fig. 1A). As such, the substrate‐binding residues in the lid region are of interest to mutate, to unravel their role in the interaction with the entering substrate and consequently their crucial impact on the enzyme activity.

In this study, Ala89 and Phe95 were assigned as interesting amino acids for mutation and further investigation of the importance of the size and polarity of alternative residues on the substrate selectivity. Ala89 is positioned at the center of the lid‐helix, whereas Phe95 is located at the end of the lid region and the end of the substrate‐binding crevice (Fig. 1B). Subsequently, seven single site‐directed mutations at the two positions (A89W, A89F, A89I, F95A, F95I, F95W, and F95Y) were introduced, and the resulting variants were produced accordingly. Ala89, located on the middle position of the lid region, was respectively mutated into Trp (A89W), Phe (A89F), and Ile (A89I) to reveal the effect of spatial hindrance toward substrate selectivity by introducing amino acid residues of different bulkiness (size contribution). Phe95, located at the end of the lid region and substrate‐binding crevice, was mutated into Ala (F95A) and Ile (F95I), respectively, to reduce the bulkiness of the side chain to different extents. In addition, aromatic residues with different properties were introduced: Trp (F95W) to increase the spatial hindrance and Tyr (F95Y) to change the polarity of the residue.

**Fig. 2:**
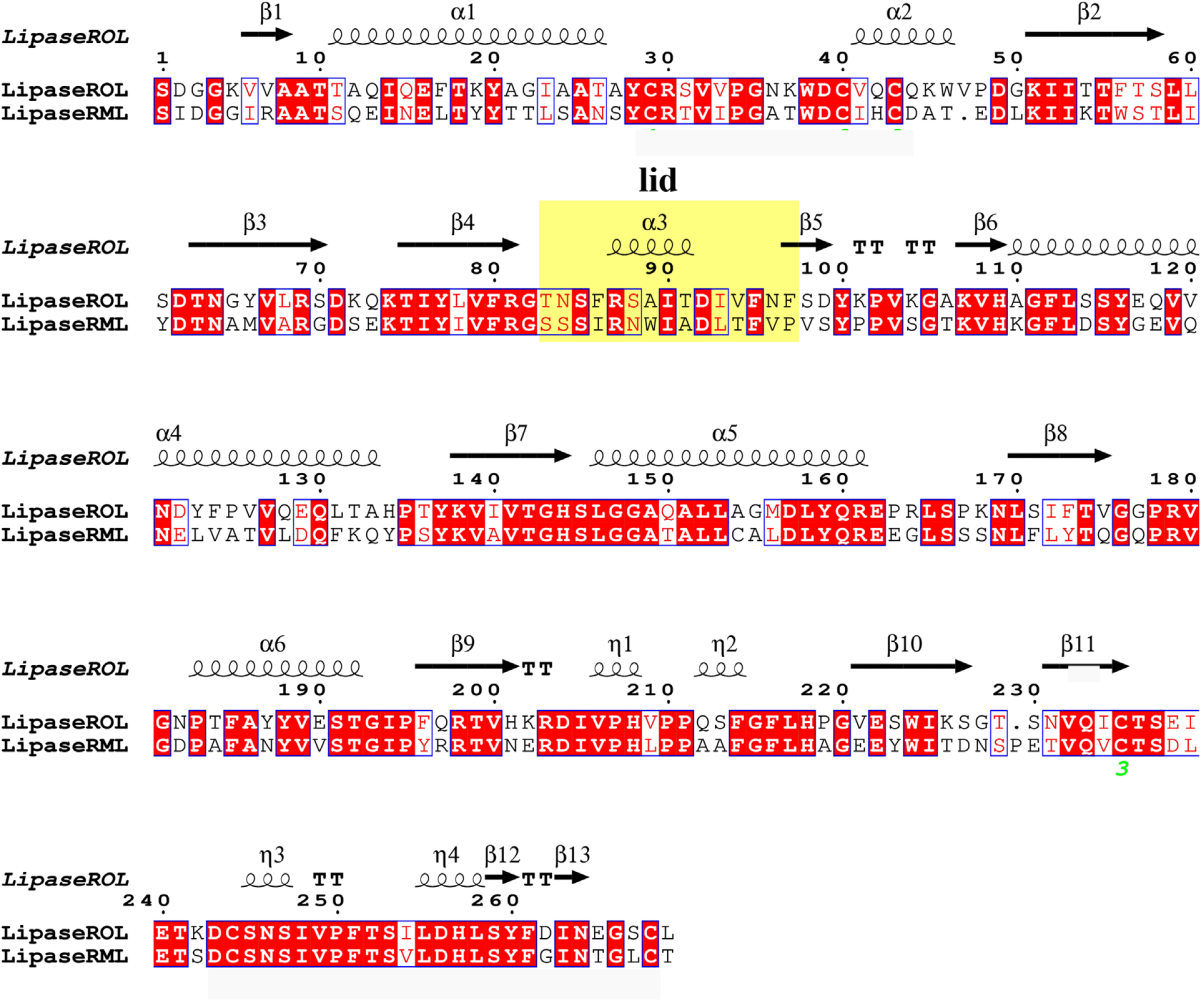
Pairwise sequence alignment of truncated lipase from *Rhizopus oryzae* (ROL) and lipase from *Rhizomucor miehei* (RML), without signal peptide and pro‐peptide. The lid region is composed of an α3 helix and flanking hinges as highlighted in yellow and marked as lid in the figure. The two enzymes share an overall 44% sequence identity, in which the lid region shares 33% sequence identity. The total identical region of sequence was marked in red while the region that shares similar amino acids (e.g., leucine and isoleucine) was marked in a box without highlighting color.

To further investigate the importance of the lid region for substrate binding, the complete 15‐residue lid region encoded in the homologous lipase RML (which shares 33% sequence identity) was introduced at the position corresponding to the original lid of ROL, completing a lid‐swap. The sequence alignment between ROL and RML, with the lid region highlighted, is shown in Fig. 2.

### MD simulations of ROL WT and variants

**Fig. 3:**
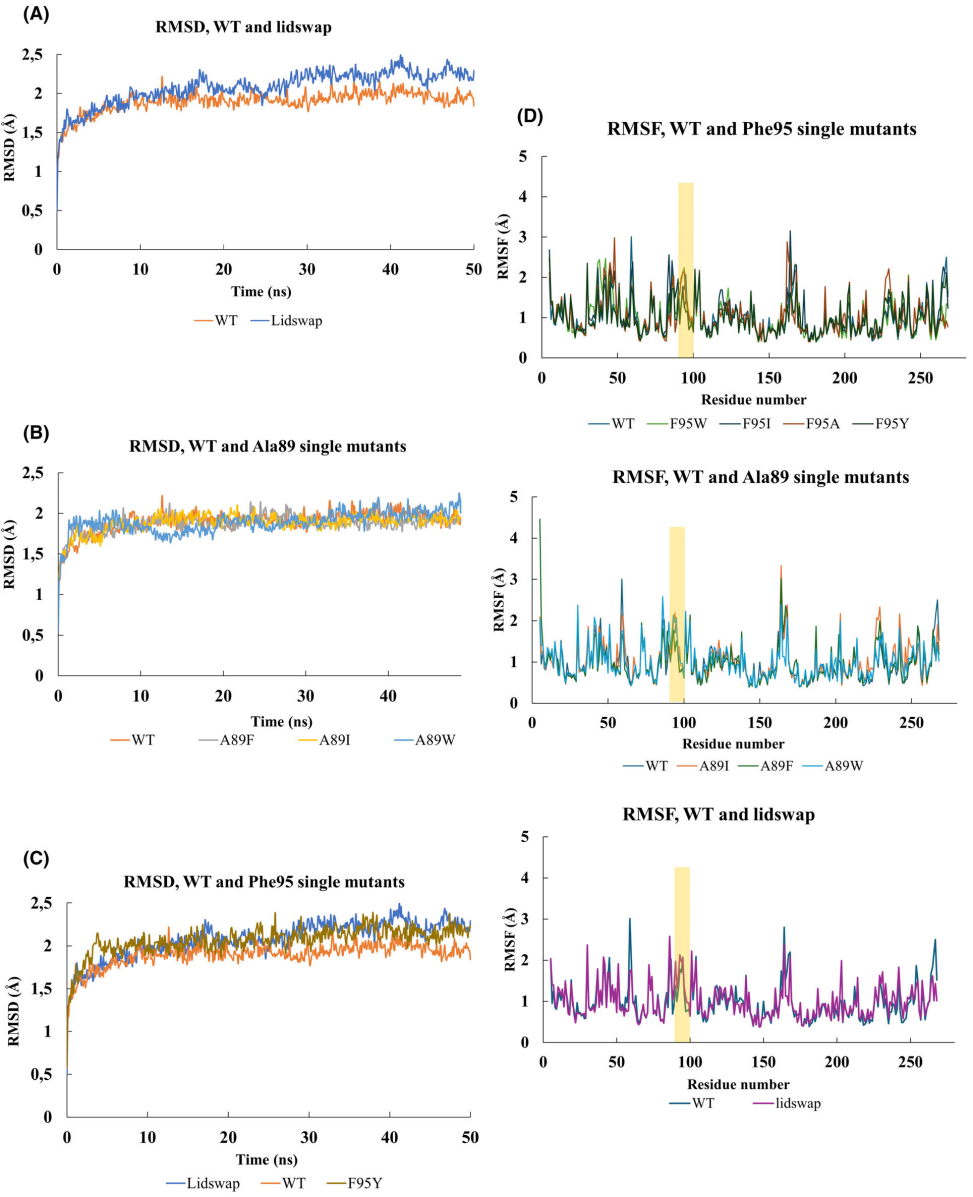
Root‐mean‐square deviation of all heavy atoms (RMSD_all_) of different ROL variants, based on all atoms in the molecules composing the enzyme, was generated during 50 ns molecular dynamic (MD) simulation with YASARA. (A) RMSD_all_ is a comparison of wild‐type (WT) (orange curve, the same in other plots) and mutant enzymes with Phe95 (F95A: green curve; F95W: brown curve; F95I: gray curve; F95Y: yellow curve) residue single mutated during the 50 ns MD simulation. (B) RMSD_all_ is a comparison of WT and mutant enzymes with Ala89 (A89F: gray curve; A89I: yellow curve; A89W: blue curve) residue single mutated during the 50 ns MD simulation. (C) RMSD_all_ comparison of WT and lidswap (blue curve) chimera mutant during the 50 ns MD simulation. (D) Root mean square fluctuation (RMSF) of different lipase from *Rhizopus oryzae* (ROL) variants. The amino acid residues composing the lid region are highlighted in yellow. The simulations were done as duplicate.

The effect of the introduced single mutations and of the lid swapping on the structural stability of the respective enzyme variant was evaluated by applying molecular dynamics (MD) simulations in a water environment. The average root‐mean‐square‐deviation (RMSD) of all atoms in the ROL WT molecule, and in all mutated variants are shown in Fig. 3A–C. The RMSDs for the WT, and all the single mutated variants, except F95Y, were stabilized at 2 Å during a 50 ns MD, while the average RMSD for the variant F95Y and lid‐swap chimera was stabilized at 2.1 and 2.2 Å, respectively (Fig. 3A,B,C). These results suggest that neither of the single mutations nor the complete exchange of the lid caused any significant structural disturbance. A larger RMSD was expected for the lid‐swap chimera, due to the combined 15‐residue mutation, but the increase in RMSD could be judged as marginal despite the larger change, indicating a good stability of the structure. The root‐mean‐square fluctuations (RMSF) also revealed that the lid‐region is located in an area with higher RMSF in the enzyme (Fig. 3D), and this is judged to be related to function, as no apparent change is obvious between the WT and the different mutated variants.

### Docking of FA ligands and MD simulation of the docked complexes

**Fig. 4:**
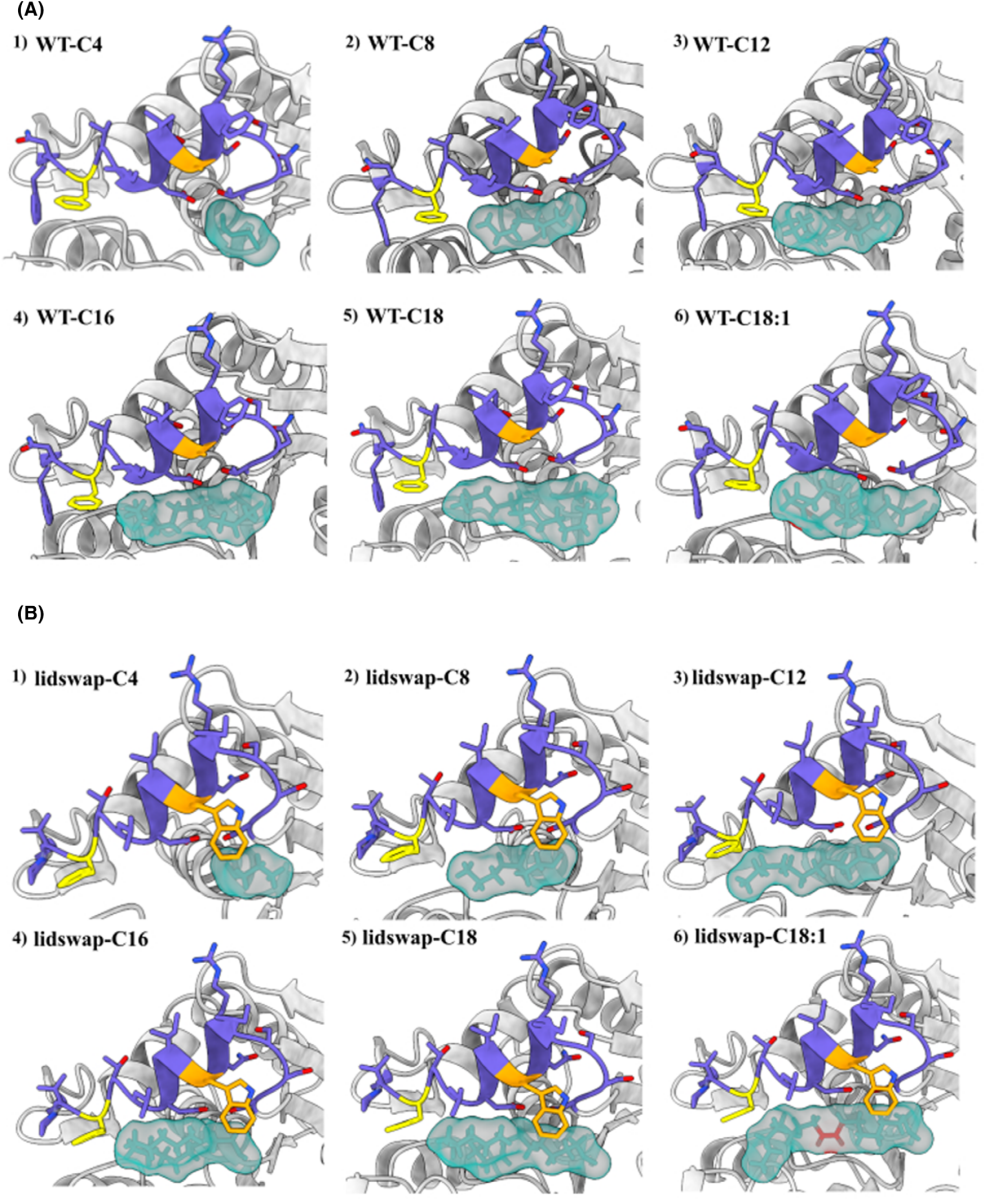
Fatty acid (FA) complex with different fatty acid ligands covalently docked in the open‐lid conformation in lipase from *Rhizopus oryzae* (ROL) wild‐type (WT) and lidswapped structure modeled using YASARA. The complexes of ROL WT are shown in panel A, while the complexes of the lidswap mutant are shown in panel B. The FA ligands docked in the models are respectively: (1) Butyrate (C4); (2) Octanoate (C8); (3) Laurate (C12); (4) Palmitate (C16); (5) Stearate (C18); (6) Oleate (C18:1). The lid region is marked in purple. Phe95, located at the end of the lid (and end of the substrate binding region), is shown in yellow. Ala89 (in WT) or Trp89 (in lidswap), which is located in the middle of the lid pointing into the substrate binding region, is shown in orange. The docked ligands are shown in cyan in surface representation. The C‐C double bond located between C9 and C10 in oleate is shown in red. Among the FA ligands with different lengths, mid‐chain fatty acids (MCFA) (C_8_ and C_12_) showed the best spatial fitting to the substrate‐binding crevice. C_4_ and C_8_ showed a similar binding pattern when docked in the WT and the lid‐swapped structure, respectively, while the longer FA ligands showed a more extended binding pattern in the lid‐swapped structure. The structures were visualized with chimerax 1.7.1 (https://www.rbvi.ucsf.edu/chimerax).

**Fig. 5:**
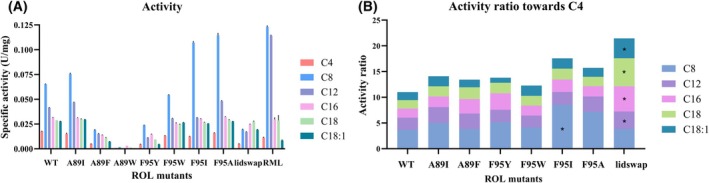
Activity profile of lipase from *Rhizopus oryzae* (ROL) wild‐type (WT), the mutated ROL variants, and lipase from *Rhizomucor miehei* (RML). The substrates used were *p*‐Nitrophenyl fatty acids (*p*NP‐FAs). (A). The specific activities of the produced variants and RML on *p*NP‐FAs of different lengths (or saturation). (B). Activity ratio of ROL WT and mutants. The activity on *pNP‐mid‐chain* fatty acid (MCFA) and long‐chain fatty acid (LCFA) esters is divided by the activity on *p*NP‐C4. This gives an indication of the substrate selectivity of ROL variants for fatty acids of different lengths. The mutated variants that showed the highest change in substrate selectivity of different *p*NP‐FAs over *p*NP‐C4 are marked with “*”. Standard deviation is shown as error bars (*n* = 3).

Six FA ligands with different lengths and saturation (C_4_, C_8_, C_12_, C_16_, C_18:0_, and C_18:1_) were docked covalently into the open‐lid structure model of the ROL WT (Fig. 4A) and the lid‐swap chimera (Fig. 4B) to investigate the effects of the lid swapping on the ligand‐binding pattern. Among the selected FA ligands, the length of the substrate‐binding crevice of the WT of ROL provided the optimum binding space for the C_8_ ligand (Fig. 4, panel 2). To fit the longer FA ligands into the substrate‐binding crevice, it was necessary to introduce bending of the carbon chain backbone (Fig. 4, panel 3–5). The longer FA ligands showed a more extended binding pattern to the lid‐swap variant (Fig. 4B), as compared to the binding pattern in the WT (Fig. 4A). This effect was also visible in the biochemical data where the selectivity of lid‐swap variant on long‐chain fatty acid esters (LCFA) was relatively higher (see below, Fig. 5B). Specific effects imposed by the single mutations on substrate binding were negligible from the docking results, given the single residue alteration (data not shown). However, mutations of Residue 89, located in the middle of the crevice, resulted in changes in the overall activity level of the enzyme (below, Fig. 5A).

The dissociation constant (*K*_d_) of docked complexes, WT‐C8, WT‐C16, WT‐C18:1, and lidswap‐C8, lidswap‐C16, lidswap‐C18:1, were extracted from the docking result and listed in Table 1. By swapping the lid region of ROL to the RML lid‐region, the *K*_d_ toward long‐chain saturated fatty acid was four times increased (C16) and two times increased (C18:1) toward the respective long‐chain unsaturated fatty acid. The *K*_d_ of the lid‐swapped enzyme also increased about 10% toward the mid‐chain fatty acid.

**Table 1:** The dissociation constant of ROL WT‐ and lid‐swap‐fatty acids complexes, extracted from YASARA.

μm	WT	Lidswap
C8	380	420
C16	17	70
C18:1	17	39

**Fig. 6:**
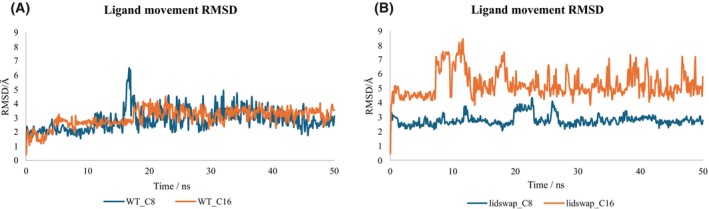
Ligand movement root mean square deviation (RMSD) of complexes of mid and long chain fatty acids with ROL WT and lidswap during 50 ns molecular dynamic (MD) simulation. The data of wild‐type (WT) complexes are shown in panel (A) while the data of lidswap complexes are shown in panel (B). The movement RMSD of C8 (blue curve) and C16 (orange curve) shows similar value in WT complexes, while lidswap‐C16 showed on average 2 Å higher ligand movement RMSD than lid‐swap‐C8. The simulations were done as duplicate.

To further investigate the interaction between mid‐ and long‐chain fatty acids with ROL WT and lidswap, 50 ns MD simulations were performed on WT‐C8, WT‐C16, lidswap‐C8, and lidswap‐C16. The ligand movement during the respective MD simulation was evaluated by analysis of ligand movement RMSD (Fig. 6). The movement RMSD of C8 and C16 shows similar value in WT complexes, while lidswap‐C16 showed on average 2 Å higher ligand movement RMSD than lidswap‐C8.

### Production and purification

The WT and mutated variants of ROL were all produced in *Y. lipolytica* in minimal medium YNBDLE, after transforming the ROL production cassette with the erythritol‐inducible promoter pEYL5AB and URA3Ex selection marker, as described previously [12]. Due to the simple composition of YNBDLE, the purification process of ROL was simplified to a single‐step dialysis (with a 25 kDa cutoff membrane in 50 mm TP buffer pH 6.8) followed by protein concentration using a 10 kDa cutoff column. The final protein concentration of all ROL variants was around 0.15 mg·mL^−1^.

### Biophysical and biochemical characterization

#### Temperature stability measurement

To reveal the impact of the introduced mutations (single mutations and lid‐swap mutation) on the thermostability, the *T*_m_ of the WT, the respective enzyme variant and a commercial RML (whose lid region was selected for the lidswap) were analyzed by high‐throughput nano‐DSF using the Prometheus NT48 system (Table 2). The resulting *T*_m_ values showed that the mutations introduced at position 95, at the end of active site cleft (Figs 1 and 4), led to negligible change of the *T*_m_ (Δ*T*_m_ < 1 °C, Table 2), and the slight increase in RMSD for F95Y in the structure model was not resulting in any decrease in thermostability. At Position 89 in the middle of the cleft, where a larger aromatic residue was introduced [A89W (1.1 °C higher) and A89F (2.1 °C higher)] (Figs 1 and 4), a slight increase in *T*_m_ was observed, potentially due to the introduction of stabilizing interactions. The reference RML showed a slightly higher *T*_m_ (2.7 °C) than ROL WT. Finally, the 15‐residue lidswap, did result in a decreased *T*_m_ (Δ*T*_m_ = −3.8 C), however, despite the relatively large number of exchanged residues, the change in *T*_m_ was not extensive, indicating that crucial interacting residues were kept by conserved amino acids. Indeed, five of the 15 residues (including Phe95) are conserved between the ROL and RML lid, while four additional residues have similar properties (Fig. 2). However, the remaining six residues imposed more significant differences in properties (including Ala89) between the lids of the respective enzyme.

**Table 2:** Melting temperatures of ROL variants at pH 6.8.

ROL variant	*T*_m_ ^a^ (°C)	∆*T*_m_ ^b^ (°C)
WT	49.8 ± 0.1	–
F95A	50.5 ± 0.2	0.7
F95Y	50.6 ± 0.2	0.8
F95I	50 ± 0.1	0.2
F95W	49.4 ± 0.1	−0.4
A89W	50.9 ± 0.1	1.1
A89F	51.9 ± 0.3	2.1
A89I	49.3 ± 0.2	−0.5
lidswap	46.0 ± 0.1	−3.8
RML	52.5 ± 0.1	2.7

^a^ *T*_m_ was measured in 50 mm phosphate buffer.

^b^ ∆*T*_m_ = *T*_m_ − *T*_m (WT)_.

Overall, the thermostability data indicate that crucial interactions for function were kept, resulting in a relatively limited change in thermostability, maintaining it within a favored temperature range for industrial processes.

The thermostability of the mutated variants were also evaluated under acidic (pH 3.4) and alkaline (pH 8.0) conditions (Table 3) and compared to the data collected at neutral pH (pH 6.8). The results depicted that the WT and all variants showed highest thermostability at pH 6.8. The F95W variant and the variants mutated at Position 89 were also less thermostable under acidic than under alkaline conditions, which could be due to the high isoelectric point of ROL (WT pI = 8.14) together with the bulkiness of the sidechain of the substituted amino acid. In all cases, the bulkiness (the size) of the substituted amino acid imposed reduced stability. This impact was most pronounced for F95W (which also displayed a somewhat reduced stability at neutral pH) and for the A89F and A89I variants (where the latter had a reduced stability at all tested pHs). An exception was the lid‐swapped variant, which appeared less sensitive to variations in pH.

**Table 3:** Comparison of melting temperature under acidic (pH 3.4), alkaline (pH 8), and neutral (pH 6.8) conditions.

ROL variant	∆*T*_m_ ^a^ (°C)	∆*T*_m_ ^b^ (°C)
WT	−4.4	−3.5
F95A	−4.2	−4.3
F95Y	−4.4	−4.4
F95I	−4.5	−4.1
F95W	−7.2	−4.1
A89W	−4.5	−3.4
A89F	−6.3	−4.0
A89I	−6.0	−4.7
lidswap	−3.0	−3.0

^a^ ∆*T*_m_ = *T*_m,pH 3.4_ − *T*_m,pH6.8_.

^b^ ∆*T*_m_ = *T*_m,pH 8.0_ − *T*_m,pH6.8_.

#### Chain length specificity of ROL variants

The activities of all the seven single mutated ROL variants, the lid‐swap chimera and the WT were analyzed, using *p*NP‐FAs esters of different chain lengths as substrates. The selected *p*NP‐FAs esters included the short‐chain fatty acid (SCFA) ester *p*NP‐C_4_, the mid‐chain fatty acid (MCFA) esters *p*NP‐C_8_ and *p*NP‐C_12_, and the long‐chain fatty acid (LCFA) esters *p*NP‐C_16_ and *p*NP‐C_18:0_ as well as the unsaturated LCFA ester *p*NP‐C_18:1_. In Fig. 5A, the specific activity on the respective substrate is displayed, and, the *v*_%_ (specific activity for the respective FA over the specific activity for the C4 substrate, Eqn 1) is shown in Fig. 5B and Table 4. The *v*_%_ change toward various FA substrates of ROL mutants compared to the WT calculated using Eqn (2) is depicted in Table 5.

**Table 4:** Activity ratio values showing the specific activity toward *p*NP‐FA over *p*NP‐C_4_ of ROL. *v*_%_ is calculated according to Eqn (1) in the Materials and Methods section. The unit of *v*_%_ is 1.

ROL variant	Activity ratio *v*_%_				
	*v*_%‐C8/C4_	*v*_%‐C12/C4_	*v*_%‐C16/C4_	*v*_%‐C18:0/C4_	*v*_%‐C18:1/C4_
WT	3.68	2.35	1.78	1.63	1.56
A89I	5.00	3.09	2.05	1.99	1.95
A89F	3.82	3.03	2.81	2.29	1.48
A89W	2.0	0.67	3.56	1.44	0.44
F95Y	5.15	2.43	3.19	2.05	0.99
F95W	4.13	2.32	1.94	1.88	2.01
F95I	8.54	2.50	2.41	2.12	2.02
F95A	7.15	3.00	2.01	1.83	1.72
lidswap	3.85	3.38	4.91	5.47	3.83

**Table 5:** Selectivity (*S*_FA‐variant_) toward a certain FA ligand. The selectivity of ROL variants toward different FAs was calculated based on Eqn (2) in the Materials and Methods section.

ROL mutants	Selectivity (%)				
	*S*_‐C8_	*S*_‐C12_	*S*_‐C16_	*S*_‐C18:0_	*S*_‐C18:1_
WT	0	0	0	0	0
A89I	36	31	15	22	25
A89F	4	29	58	40	‐5^a^
F95Y	40	3	79	26	‐36^a^
F95W	12	‐1^a^	9	15	29
F95I	132	6	35	30	29
F95A	94	28	13	12	10
lidswap	5	44	176	236	145

^a^ A minus value means the selectivity of the variant is lower compared with WT toward the substrate (Eqn 2).

Comparison of the activity ratio (*v*_%_) for different FA‐esters showed that most of the Phe95 mutated variants displayed highest substrate preference toward MCFA‐esters (Table 4). They also displayed a higher selectivity toward MCFA‐ (C_8_ and C_12_) and LCFA‐esters (C_16_, C_18:0_ and C_18:1_) than the SCFA (C_4_), with a generally higher selectivity for C_8_, when compared with WT ROL (Fig. 5 and Table 5, column *S*_C8_). This was especially pronounced when the aromatic sidechain was removed. (Table 5, column *S*_C8_). Alteration of Phe95 to Trp (F95W) did not result in any specific change in the activity profile but resulted in a small reduction in thermostability (Tables 2 and 3). Introduction of the more polar Tyr‐residue (F95Y), was not beneficial for the overall specific activity, which was reduced toward all *p*NP substrates compared with the WT, but in this case, selectivity changed, and this variant was more selective toward the MCFA‐ (*p*NP‐C_8_) and LCFA‐esters (*p*NP‐C_16_ and C_18_) compared to the SCFA‐ester C_4_ (Fig. 5B and Table 5). The unsaturated *p*NP‐oleate C_18:1_ was however not preferred and displayed a 36% selectivity reduction, as compared with WT (Fig. 5B and Table 5).

Mutagenesis of Ala89 generally led to a reduced specific activity (Fig. 5A), possibly due to clashes with neighboring residues (Fig. 5B and Table 5), except for the A89I variant. The A89I variant displayed a similar selectivity profile compared to WT (Fig. 5A), but with a relative increase in specific activity toward C_8_ (36% higher), C_12_ (31% higher), C_16_ (15% higher), C_18:0_ (22% higher), and C_18:1_ (25% higher) (Table 5), showing that the relative activity on C_4_ was reduced. A89W lost most of the activity toward *p*NP‐FAs of all selected chain lengths, and A89F also showed a significant loss of specific activity toward all target substrates (ranging from 55% to 70% loss compared with WT), showing that this single mutation led to a too large change in side‐chain bulkiness. In this case, the preference for MCFA‐esters was lower than for A89I, while A89F increased the preference for the saturated LCFAs. Hence, both A89I and A89F resulted in improved selectivity for mid‐chain fatty acid esters (C_8_ and C_12_) while A89F also showed a higher preference for LCFAs (C_16_ and C_18_) in comparison with the WT, indicating that Residue 89 has an influence on substrate accommodation. However, despite the relative increase, the Ala89 mutants still showed the highest preference toward MCFA‐esters when comparing the activity ratio *v*_%_ toward different FA‐esters of the mutants (Table 3).

The lid‐swapped variant, with the lid region replaced by the lid region of the RML homolog, showed slightly higher selectivity toward MCFA‐esters than C_4_ but with significantly higher selectivity toward LCFAs [C_16_ (176% higher), C_18:0_ (236% higher), C_18:1_ (145% higher)] (Fig. 5B, Table 5). This was mainly caused by a significant reduction in specific activity on the shorter substrates (Fig. 5A). What is especially interesting is that the lid‐swapped enzyme variant tuned the substrate preference of ROL toward LCFAs despite the higher activity toward MCFAs shown for both ROL WT and RML, which has not been reported previously (Table 4).

## Discussion

Lipases (EC3.1.1.3) are extensively used enzymes to modify the structure of lipids to meet special needs for several industrial applications, for example, food, health and nutrition, and personal care [15]. The broad application of lipases leads to an interest in their engineering to boost or alter their specific activity toward different fatty acids in lipids. In this work, mutations were introduced in a region, termed the lid, of the lipase from the filamentous fungus *R. oryzae* (ROL) to study the effect on substrate selectivity. In *R. oryzae*, ROL is first synthesized in a precursor form, with a 26 amino acid presequence, followed by a 97 amino acid prosequence attached to the N terminus of a 269 amino acids mature sequence [11]. The overall organization of the enzyme is structurally homologous to the *Y. lipolytica* extracellular lipase Lip2 (despite differing amino sequences especially in the pre‐ and proregions), making *Y. lipolytica* an interesting host for recombinant production [12]. Efficient production of ROL was also accomplished in the *Y. lipolytica* system [12], allowing molecular development of the enzyme variants reported here, that was targeting residues proposed to influence the activity profile of the enzyme. ROL has, like most lipases, a lid domain that covers the catalytic triad, and when the α‐helical lid moves at a lipid–water interface, it creates a large hydrophobic patch around the catalytic triad, activating the lipase. The open‐lid structure model of ROL, generated in a previous work [15], was used to select target residues (Ala89 and Phe95) and allowed bioinformatics analysis of the effect of mutagenesis on the structure, which showed that the proposed mutations would likely not damage structural stability. Even introduction of the complete lid from the related RML enzyme, involving a 15‐residue change (Residues 83–97, of which five including Phe95 were completely conserved), resulted in maintained structural stability, and production of an active enzyme‐variant in the *Y. lipolytica* system.

When changing Phe95 to amino acid residues with smaller sidechains, including Ile (F95I) or Ala (F95A), the selectivity toward MCFAs and LCFAs increased compared with the SCFA C_4_ (Table 4). This can be explained by the reduction in the side chain size that slightly expands the space in the substrate‐binding crevice, allowing the longer chain fatty acid to extend and be better accommodated, thereby enhancing the activity toward longer fatty acid ligands. However, this appears to occur within a limited FA‐range, as the enhancement toward LCFAs was less significant than for MCFAs, indicating that the general size of the substrate binding region still fits MCFAs the best (Fig. 4). When mutating Phe95 to a more polar residue (Tyr), the variant again showed an increased activity for MCFAs and LCFAs (compared to C_4_) but not when the substrate was unsaturated (i.e., oleic acid instead of stearic acid), which is aligned with previous research [20]. This could be due to the introduction of the ‐OH group on Tyr, which could affect the interaction with the C=C double bond.

In addition to Phe 95, Ala89 was also targeted. Ala89 is located in the center of the lid pointing toward the entrance of the substrate‐binding region (Fig. 1B). Interestingly, in the RML homolog, the corresponding residue is not conserved, and the corresponding residue in RML is Trp [15]. Mutation of Ala89 into a Trp (or Phe) however led to major activity losses against all *p*NP‐FA substrates, showing that this change must be complemented by additional changes in neighboring residues (as in the lid‐swapped variant) to create sufficient space for maintained activity. As reported previously, loss of half the activity on triolein has been reported, after making a similar single residue change (A89W) in line with our result [21]. Hence, the single introduction of the Trp (or Phe) in the lid region of ROL on Ala89 position, results in a too big spatial hindrance colliding with the neighboring residue Phe86 (where RML, and the lid‐swapped variant has Ile at the corresponding position). In the single mutant (A89W), the change also negatively affected structural stability of the α‐helix composing the lid, which was observed with the trajectories during the MD simulation. Furthermore, with both Trp89 and Phe86 located closely together pointing toward the entrance of the substrate binding region, the spatial hindrance of substrate entering and leaving the catalytic center is increased, which may explain the general decrease in enzyme activity toward all selected FAs.

**Fig. 7:**
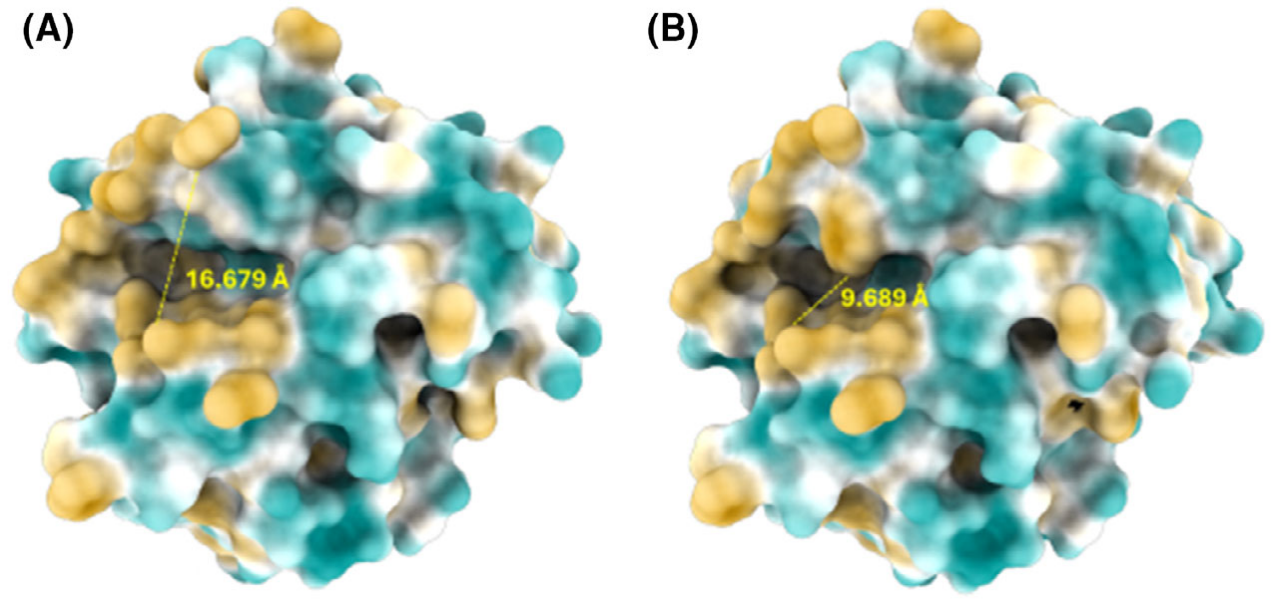
Structural model (surface hydrophobicity representation) of lipase from *Rhizopus oryzae* (ROL) wild‐type (WT) and lidswap mutated variant. The model of WT is shown in panel (A) while the model of lidswap mutant is shown in panel (B). The region that is colored as turmeric yellow indicates that it shows hydrophobic properties. The region that is colored in turquoise green shows hydrophilic character and the white region is neutral. This shows that the lid swapping increased the hydrophobic area on the top part of the substrate binding crevice due to the Trp89 located on the inner side of the swapped lid of lipase from *Rhizomucor miehei* (RML). The lid swapping also added spatial hindrance for substrate entering and leaving the substrate binding crevice. The Trp89 (Ala89 on WT ROL) on the lid‐swapped variant reduced the size of the substrate entrance from 16.679 to 9.689 Å. The structures were visualized with chimerax 1.7.1 ( https://www.rbvi.ucsf.edu/chimerax ).

Due to the essential function of the lid region, the lidswap (replacing the ROL lid with the lid from the related RML enzyme) was shown to significantly influence lipase substrate selectivity. A corresponding approach has been made by Yu et al., who swapped the lid of an esterase (introducing a smaller and more hydrophobic lid) to a lipase from *Rhizopus chinensis* (RCL), and by this transferred RCL into an esterase, showing the importance of the lid for substrate specificity [18]. In this work, to minimize structural perturbation, we selected a closely related enzyme, RML, for lid swapping to ROL. These enzymes are structurally similar (33% sequence identity) and have lids of the same size. The introduction of the lid of RML into ROL resulted in higher substrate selectivity toward LCFAs, compared to the WT. The lid swapping also added the spatial hindrance during substrate entering and leaving the substrate‐binding crevice, as the RML has a Trp at the identical location of the Ala89 in the lid of ROL. This results in significant size reduction in the substrate entrance from 16.679 to 9.689 Å (Fig. 7). This may have been thought to introduce additional spatial hindrance upon fatty acid entering and leaving the substrate binding site. The opening distance of lid of ROL WT, lidswap, and their complexes with C8 and C16 was screened during the 50 ns MD simulation with comparison every 100 ns (Table 6). By comparing the standard deviation of the lid opening distance of free WT and lidswap, the result indicates that the lid on lid‐swap variant showed a higher flexibility compared to WT. C16 ligand shows a stabilization of the lid for both enzyme variant while C8 shows stabilization only toward lidswap. The lid of WT‐C8 complex showed slightly higher flexibility compared to WT and WT‐C16, while lidswap‐C8 showed a similar lid flexibility compared to lidswap‐C16. This similarity is correlated with the activity profile of WT and lidswap toward MCFA and LCFA. This result may indicate that the activity of the ROL variant toward different fatty acid is also correlated with the flexibility of the lid region when the fatty acid ligand interacts with the active site, and the discovered trend in this study indicated a positive correlation.

**Table 6:** Lid opening distance (Å) of ROL WT‐ and lid‐swap‐fatty acids complexes during 50 ns MD simulation.

	0 ns	100 ns	200 ns	300 ns	400 ns	500 ns	std^a^
WT	16.67	11.42	12.88	9.95	9.06	9.63	2.60
WT‐C8	13.06	10.26	10.25	7.94	4.56	6.17	2.83
WT‐C16	12.38	13.62	12.07	11.92	11.47	9.27	1.31
lidswap	10.25	6.96	8.79	14.18	13.08	15.26	2.98
lidswap‐C8	9.27	9.69	12.30	11.35	14.91	12.40	1.88
lidswap‐C16	9.34	9.34	9.89	11.28	12.89	12.13	1.38

^a^ Standard deviation of the lid opening distance of the 5 time points.

The result from the MD simulation of docked complexes between fatty acid ligands and ROL WT or the lid‐swap variant indicated a three times higher *K*_d_ between C16 and the lid‐swap variant compared to WT (Table 1). The ligand movement RMSD of lidswap‐C16 also indicates a larger movement of C16 when docked in the lid‐swap variant compared to when docked in WT. It has been shown in previous research that LCFAs may inhibit the lipase by binding too well (low *K*_d_) [22], reducing the enzymes´ general turnover rate. Therefore, by combining the substrate selectivity result with the bioinformatics, we may interpret the result as a weakened binding between LCFAs and the lid‐swap variant, which reduced the inhibition between ROL and LCFAs and subsequently increased the general activity toward LCFAs. However, the introduction of the single bulkier residue of the RML lid region increased the hindrance of long fatty acid substrates to enter, which resulted in a more significant effect on MCFAs. The swapping of the lid region also suggests an expansion of the hydrophobic patch residing on the top of the substrate‐binding crevice due to presence of a Trp residue located on the lower side of the RML lid, which may enhance the hydrophobic interactions between the fatty acid ligand and substrate‐binding crevice that may promote alternating interactions that allows the fatty acid to be maintained in the substrate‐binding crevice.

In addition to the lidswap made in the current work, the RML lid has also been introduced in the lipase RCL [18]. The previously reported swapping of the RML‐lid to RCL led to an increase of the activity toward triglyceride products (C_2_, C_6_, C_8_, C_12_ and C_16_) from 1.5‐ to 3.3‐fold but a 40% decrease of tristearin activity at 40 °C [18]. RML has been reported by Berger et al. to reveal highest activity toward C_8_ triglycerides followed by C_16_, C_18_, C_10_, C_12_, with negligible activity against triolein [23]. However, RML also revealed the highest selectivity toward oleic acid monoglycerides [24]. The contradicting results from the two reports might be due to different reaction conditions and types of substrates. In this study, our activity assays were conducted with simple *p*‐nitrophenyl esters of different fatty acids with the same molar concentration under 30 °C, with 10% V/V 1‐propanol for solubilization of all substrates, to evaluate our mutants. Under this condition, our result indicates that RML showed the highest activity toward C8 > C12 > C16 > C18 > C4 > C18:1, which is similar to ROL. Nevertheless, these data show that the lid is fundamentally important for selectivity and may be targeted to introduce changes in the activity profile of lipases. However, only the lid region does not determine the selectivity, it is the combination of the lid and the other region of the active site that is essential for substrate selectivity. In conclusion, the lid region apparently has a complex role in lipase function, including both substrate selectivity and structural stability.

## Conclusion

In this research, single residue mutated ROL variants targeting Ala89 or Phe95 located on the lid region of ROL were constructed. Furthermore, a lid‐swapped 15 residue chimera variant was constructed, introducing the lid region of the homologous lipase RML into ROL. The designed variants were produced and characterized to evaluate the effect on substrate selectivity. The results confirmed that the lid region of ROL is highly relevant for fatty acid selectivity. Mutation of Phe95 located at the end of the lid‐ and substrate‐binding region to a smaller residue significantly increased the enzyme selectivity toward MCFA (especially C8) over SCFA (C4) and LCFAs. A drastic increase in the side chain size at Position 89 (from Ala89 to Phe or Trp) resulted in a strong general reduction in enzyme activity but also changed the activity profile toward longer FAs. However, by swapping the lid region of ROL with RML (which included Trp at the position corresponding to Ala89), the substrate preference of ROL was more effectively tuned toward LCFAs (from MCFAs), which has not been reported previously. The generated variants of this work subsequently demonstrate potential to tailor enzyme activity of lipases toward the production of structured lipids. The mutagenesis did not result in significant changes in the thermostability of the enzyme, which maintained reaction conditions, allowing the required working temperature for use in the fat and oil industry.

## Materials and methods

### Structure modeling and molecular dynamics simulations

The structural models of open‐lid ROL wild‐type (WT) and mutants were generated using SWISS‐MODEL (https://swissmodel.expasy.org/) with the open‐lid lipase structure from *Rhizomucor miehei* (PDB:4TGL, sequence similarity 47%) as template [25]. Modeling was followed by molecular dynamic (MD) simulations and substrate docking performed with the yasara program (Version 19.12.14) [26]. Sequence alignment between matured ROL and matured RML was made with the Escript 3.0 server (https://espript.ibcp.fr/ESPript/cgi‐bin/ESPript.cgi) [27].

MD simulations of ROL mutants were run with an internal YASARA *md_run* macro with water as the solvent. Each simulation was run for 50 ns with a NaCl concentration of 0.9% to neutralize the simulation cell at pH 6.8. To remove clashes, steepest descent and simulated annealing minimizations were applied before the simulation. During simulation, the AMBER14 force field was applied for the solute and the motions equations were integrated with time intervals of 1.25 and 2.5 fs for bonded and nonbonded interactions, respectively. The simulation cell was set as cubic with a cell extension of 10 Å on each side of the protein and a periodic boundary. The similar method has been described in our previous study with a different solvent in the simulation environment. [15].

### Docking of FA ligands

Docking procedures were executed using AutoDock within the YASARA program, employing the default docking parameters provided in the “examples” subdirectory. Initial assignment of point charges followed the AMBER03 force field [28] subsequently attenuated to replicate the less polar Gasteiger charges utilized for optimizing the AutoDock scoring function. To model a potential covalent intermediate in the enzymatic reaction between the lipase molecule and different FA ligands, stearate and oleate ligands were covalently docked into molecules, respectively, using the *dock_runcoval* macro script in the YASARA program.

The structures of the different FA ligands (corresponding to the *p*NP‐linked FA ligands used in the activity assays, below) were constructed and subjected to energy minimization with the YASARA program. A covalent bond was established between the carboxyl carbon on the respective FA ligand and the oxygen on the R‐chain of the catalytic serine (OG atom, Ser145 for ROL) in the lipase molecule. In each docking, both the lipase molecule (receptor) and the FA molecule (ligand) were treated as flexible structures. Twenty‐five docking runs of the covalently bound ligand were clustered for each FA ligand, resulting in two distinct complex conformations differing by at least 5.0 Å heavy atom RMSD after superposition on the receptor [28, 29]. The conformation with a more positive binding energy was selected. The method has been described in our previous study as well [15].

### Plasmid propagation in *E. coli*

In total, seven variants were constructed, each with a single site‐directed mutation (F95A, F95W, F95Y, F95I, A89I, A89W, and A89F) introduced in the gene encoded in the production cassette of ROL WT. In addition, a lid‐swapped chimera was constructed (where the part of the ROL gene encoding the 15 residues composing the lid is replaced by the corresponding RML part). All variants were designed to encode the signal peptide (SSL2) from *Y. lipolytica*, the propeptide region and the mature region of ROL. All variants were then synthesized by Biocat (BioCat GmbH, Heidelberg, Germany) and codon‐optimized based on the preference of *Y. lipolytica*. They were then cloned into the pET3a vector with ampicillin resistance utilizing the *Bam*HI‐*Avr*II restriction enzyme digestion sites. The ROL expression cassette was then cut from the carrier vector pET3a with the *Bam*HI‐*Avr*II restriction enzymes and transferred into the ROL expression vector JME5579, which is designed for lipase production in *Y. lipolytica* and is containing kanamycin resistance. The synthesized plasmids were then transformed into *E. coli* DH5α competent cell (ThermoFisher Scientific, Carlsbad, CA, USA) for propagation. The *E. coli* strains were grown at 37 °C in Lysogeny Broth (LB) medium with the addition of either kanamycin sulfate (50 μg·mL^−1^) or ampicillin (100 μg·mL^−1^) as antibiotic selection marker.

### Production in *Y. lipolytica* and purification of ROL

After propagation in *E. coli*, the designed ROL expression plasmids were digested by the *Not*I restriction enzyme to release the lipase production cassette with flanking zeta sequences for integration into the *Y. lipolytica* genome, followed by *Y. lipolytica* transformation. The released lipase production cassette sequence (400 ng DNA) was transformed into *Y. lipolytica* strain JMY8647 (MatA *ura3‐302*, *leu2‐270‐LEU2‐*ZETA, *xpr2‐322*, Δ*lip2*, Δ*lip7*, Δ*lip8*, Δ*lys5*, Δ*eyk1* Δ*mhy1*) with the lithium acetate method [30]. Transformants were selected in YNBD medium with uracil as the selection marker.

To produce the ROL WT and mutated variants, precultures (5 mL) of *Y. lipolytica* cells, transformed with the gene encoding the respective ROL‐variant, were grown in minimal glucose medium supplemented with lysine (YNBDL). YNBDL contained 1.7 g·L^−1^ of yeast nitrogen base (YNB) without amino acids and ammonium sulfate (YNBww, BD Difco, Paris, France), but with 5.0 g·L^−1^ of NH_4_Cl, 50 mm Tampon phosphate (TP) buffer (Na_2_HPO_4_·2H_2_O, KH_2_PO_4_, pH 6.8), 5 g·L^−1^ of glucose (D) and 0.8 g·L^−1^ lysine (L), due to the auxotrophic requirement. Inducible medium YNBDLE, also containing 5 g·L^−1^ of erythritol (E) for promoter induction, was used as production medium. Cultures (25 mL) inoculated to an optical cell density at 600 nm (OD_600_) of 0.5 were grown in YNBDLE in 250 mL baffled flasks, for 72 h at 28 °C, and at 160 rpm (in triplicates). After growth, the cultures were centrifuged, and supernatants were used to collect the recombinantly produced enzymes for evaluation of the activity profile and stability of the different ROL mutants.

Prior to analysis, purification of ROL was achieved by dialysis with a 25 kDa cutoff membrane in 50 mm pH 6.8 TP buffer, followed by a 2× concentration with a 10 kDa cutoff protein concentrator (Pierce™ Protein Concentrators PES, Rockford, IL, USA). The purity of the respective protein was evaluated with sodium dodecyl sulfate (SDS)/polyacrylamide gel electrophoresis (PAGE).

### Activity assay of ROL variants using *p*‐nitrophenol‐FA substrates

The activity of the ROL variants was measured by monitoring the release of *p*‐nitrophenol (*p*NP) from different *p*NP‐FA substrates, which respectively included: 0.2 mm *p*NP butyrate (*p*NP‐C_4_), 0.2 mm *p*NP octanoate (*p*NP‐C_8_), 0.2 mm *p*NP dodecanoate (*p*NP‐C_12_), 0.2 mm *p*NP palmitate (*p*NP‐C_16_), 0.2 mm *p*NP stearate (*p*NP‐C_18:0_), and 0.2 mm *p*NP oleate (*p*NP‐C_18:1_). The reaction was performed at 30 °C, pH 6.8 and the released *p*NP molecule was monitored spectrophotometrically using a UV–visible spectrophotometer (MultiskanGo, Thermo Scientific) at A_405 nm_ (ε = 10 998 m ^−1^ cm^−1^) in a 96‐well microtiter plate. The reaction was performed at 30 °C, pH 6.8. An aliquot of the culture supernatant (10 μL) was mixed with the respective *p*NP‐FA substrate. A 2 μL 20 mm concentration of the respective *p*NP‐FA substrate dissolved in 1‐propanol were diluted 50 times with phosphate buffer pH 6.5 and 1‐propanol with a final 1‐propanol concentration of 10% (V/V). One unit of activity was defined as the release of 1 μmol *p*NP per min. All the measurements were performed in triplicates.

The fatty acid substrate preference of a specific ROL variant was assessed using the parameter activity‐ratio (*v*_%‐FA/C4_). This parameter is defined as the specific activity (*v*) of the variant toward a *p*NP‐FA substrate divided by its specific activity toward the shortest substrate, *p*NP‐C4, as outlined in Eqn (1). If a ROL variant showed a higher *v*_%‐FA/C4_ toward a specific *p*NP‐FA substrate, it is considered to have a higher preference for the specific FA as a substrate over the other FAs.

The selectivity toward a certain FA ligand among different ROL variants were evaluated by comparing their *v*_%‐FA_ toward the FA ligand. The parameter *S*_FA‐variant_ was used to compare the selectivity which is calculated as shown in Eqn (2).

(1) $$v_{\%-\mathrm{FA}/C4}=\frac{v_{p\mathrm{NP}-\mathrm{FA}}}{v_{p\mathrm{NP}-C4}}$$

(2) $$S_{\mathrm{FA}-\text{variant}1}=\frac{v_{\%-\mathrm{FA}-\text{variant}1}-v_{\%-\mathrm{FA}-\mathrm{WT}}}{v_{\%-\mathrm{FA}-\mathrm{WT}}}$$

### Thermostability measurement by nano‐differential scanning fluorimetry (nano‐DSF)

The thermostability was studied by monitoring thermal denaturation with high‐throughput nano‐differential scanning fluorimetry (nano‐DSF) in a Prometheus NT.48 instrument (Nano Temper Technologies, Munich, Germany). Samples of ROL variants and commercial RML were prepared at 4 mg·mL^−1^ in TP buffer pH 6.8 and loaded into capillaries (Nano Temper Technologies). The intrinsic fluorescence intensity ratio between 350 and 330 nm was measured over a temperature ramp of 1 °C·min^−1^ from 20 to 95 °C. The excitation power was adjusted to 40%. Each variant sample was run in triplicates. These data were processed in the program thermocontrol v.2.0.4 (Nano Temper Technology: Munich, Germany) to determine the melting temperature (*T*_m_) for each sample. A 50 mm sodium citrate buffer was used to analyze the *T*_m_ of the ROL variants at three different pHs (3.4, 6.8 and 8.0).

### Protein analysis

Pierce™ Bradford reagent (ThermoFisher Scientific, Waltham, MA, USA) was used for protein concentration determination with bovine serum albumin (BSA) as standard. Absorbance (A_595 nm_) of triplicate samples was measured at 595 nm.

**Fig. 8:**
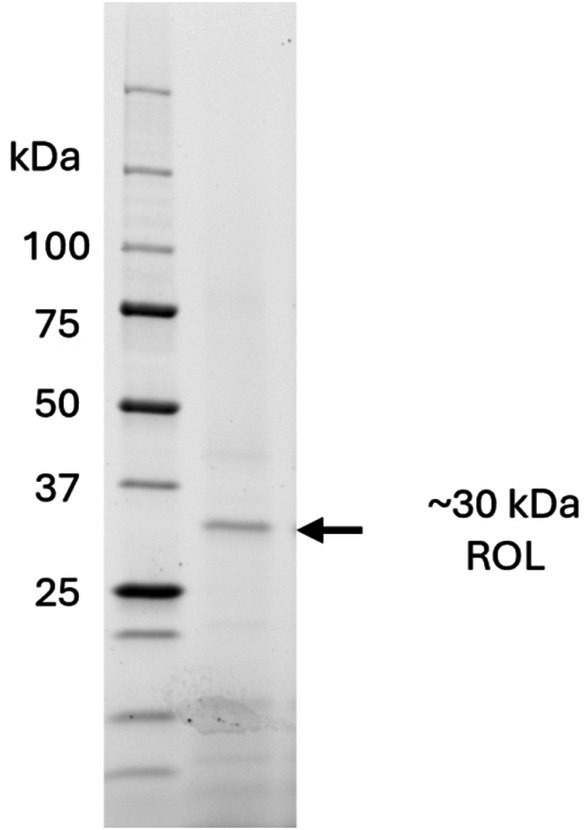
SDS/PAGE gel showing that the purified wild‐type (WT) of lipase from *Rhizopus oryzae* (ROL) has a molecular weight (MW) of 30 kDa determined using the protparam tool at the ExPASy Proteomics Server. (https://web.expasy.org/compute_pi/).

Proteins underwent SDS/PAGE using a 4%–15% Mini‐PROTEAN TGX gel (Bio‐Rad Laboratories Inc, Hercules,CA, USA) (Fig. 8). Molecular weight standards were represented by 5 μL of Precision Plus Protein Unstained Standards (Bio‐Rad Laboratories Inc). Supernatants were combined with 4× Laemmli buffer (Bio‐Rad Laboratories Inc), supplemented with 200 mm dithiothreitol (DTT), and subjected to boiling at 100 °C for 10 min.

## Conflict of interest

The authors declare no conflict of interest.

## Author contributions

ZD was involved in conceptualization, methodology, writing—original draft preparation. ENK designed the current study. ENK and MHM were involved in writing—review and editing. ENK MHM, and KO were involved in supervision. ENK and KO were involved in funding acquisition. ZD executed the enzymatic experiments and modeling and molecular dynamics of the catalytic modules. ZD and ENK designed the current study. All authors have read and agreed to the published version of the manuscript.

## Data Availability

The data that support the finding in this study can be found in the main manuscript Figs 1, 2, 3, 4, 5, 6, 7, 8 and Tables 1, 2, 3, 4, 5, 6. The underlying data for each figure are available from the authors upon request.
